# Mutant β_1_-adrenergic receptor improves REM sleep and ameliorates tau accumulation in a mouse model of tauopathy

**DOI:** 10.1073/pnas.2221686120

**Published:** 2023-04-04

**Authors:** Qing Dong, Louis J. Ptáček, Ying-Hui Fu

**Affiliations:** ^a^Department of Neurology, University of California San Francisco, San Francisco, CA 94143; ^b^Institute for Human Genetics, University of California San Francisco, San Francisco, CA 94143; ^c^Weill Institute for Neuroscience, University of California San Francisco, San Francisco, CA 94143; ^d^Kavli Institute for Fundamental Neuroscience, University of California San Francisco, San Francisco, CA 94143

**Keywords:** natural short sleep, tau pathology, Alzheimer’s disease, human genetics, neuropathology

## Abstract

Sleep is essential for our well-being, and chronic sleep deprivation has unfavorable health consequences. We recently demonstrated that two familial natural short sleep (FNSS) mutations, *DEC2-P384R* and *Npsr1-Y206H*, are strong genetic modifiers of tauopathy in *PS19* mice, a model of tauopathy. To gain more insight into how FNSS variants modify the tau phenotype, we tested the effect of another FNSS gene variant, *Adrb1-A187V*, by crossing mice with this mutation onto the *PS19* background. We found that the *Adrb1-A187V* mutation helped restore rapid eye movement (REM) sleep and alleviated tau aggregation in a sleep–wake center, the locus coeruleus (LC), in *PS19* mice. We found that ADRB1^+^ neurons in the central amygdala (CeA) sent projections to the LC, and stimulating CeA^ADRB1+^ neuron activity increased REM sleep. Furthermore, the mutant *Adrb1* attenuated tau spreading from the CeA to the LC. Our findings suggest that the *Adrb1-A187V* mutation protects against tauopathy by both mitigating tau accumulation and attenuating tau spreading.

## Significance

Age-dependent dementia is one of the largest societal and economic burdens, and the need for strategies to promote healthy aging is becoming increasingly urgent with the trend toward longer life span. Methods to mitigate age-related diseases will significantly impact future human health worldwide. Although sleep difficulties often accompany neurodegeneration, individuals with the Familial Natural Short Sleep (FNSS) trait rarely suffer from age-related diseases, despite their lifelong short sleep duration, suggesting a potential protective function of FNSS mutations. Here, we describe a FNSS mutation that lessens the development of tau pathology and REM (Rapid Eye Movement) sleep alteration in tau mice. This study supports that improving sleep quality represents a potential approach to delay the onset and progression of tauopathies.

Tau is an intrinsically disordered protein found mainly in neuronal axons (1). Abnormal phosphorylation and aggregation of tau leads to a class of neurodegenerative diseases known as tauopathies, including Alzheimer’s disease (AD), Pick’s disease, progressive supranuclear palsy, and corticobasal degeneration. AD is the most common neurodegenerative disease (2, 3). Sleep disorders are prevalent in tauopathies, and over 60% of AD patients exhibit clinical sleep disturbances (4). Moreover, growing evidence reveals that sleep disturbance usually occurs in the early stages of AD (5–8). Recent studies reported that the sleep–wake cycle regulates tau protein levels and its aggregation. One night of sleep deprivation (SD) as well as chronic sleep restriction results in increased AD pathology in the brain (9–14), confirming the intimate connection between sleep and tau pathology.

Familial natural short sleep (FNSS) is a Mendelian trait in which individuals require only 4 to 6 h of sleep each night to feel well rested. We have identified mutations in four different genes in humans with FNSS (15–18). These short sleepers typically do not desire more sleep, feel rested in the morning, and do not have any obvious health problems, suggesting that they might have better sleep efficiency/quality. We recently demonstrated that two FNSS mutations (*DEC2-P384R* and *Npsr1-Y206H*) are strong genetic modifiers of AD pathology (19). We found that these two FNSS mutations dramatically reduce tau accumulation in *PS19* mice and may regulate tau pathology through different pathways (19). This observation prompted us to investigate the impact of another FNSS mutation, *ADRB1-A189V*, on *PS19* mice. The *ADRB1* mutation changes amino acid 187 in the β_1_-adrenergic receptor (β_1_AR) from alanine to valine (16).

ADRB1 belongs to one of the subtypes of adrenergic receptors (ARs), which are G protein-coupled receptors activated by noradrenaline or norepinephrine (NE). The central noradrenergic neurons are mainly located in a small nucleus in the brainstem called the locus coeruleus (LC) and play important roles in regulating the sleep–wake cycle and neurophysiological processes (20–22). Dysfunction of the noradrenergic system has been observed in neurodegenerative diseases, such as AD. It has been reported that LC neurons are among the first neurons across the lifespan to exhibit the AD-like neuropathology characterized by hyperphosphorylated tau, and loss of LC neurons is detectable in the human brain (23). Moreover, chronic lack of sleep has been shown to advance the tau pathology in *PS19* mice and result in a significant reduction of LC neurons even in young adult mice (24, 25). These studies indicate the crucial impacts of the noradrenergic system on sleep–wake regulation and AD pathogenesis.

*PS19* mice exhibit decreased rapid eye movement (REM) sleep and severe tau pathology with increasing age (26). Therefore, to investigate the impact of *ADRB1-A189V,* we studied the tau pathology and sleep features in double-mutant mice derived from crossing *Adrb1-A187V* mice onto the *PS19* background. We observed that the *Adrb1-A187V* mutation ameliorated tau pathology in the LC and helped restore REM sleep in *PS19* mice. The *Adrb1-A187V;PS19* mice were more resistant to sleep loss-induced memory impairment than *PS19* mice. We also found that ADRB1^+^ neurons in the central amygdala (CeA) send projections to the LC and are involved in REM sleep regulation. Furthermore, the *Adrb1-A187V* mutation significantly prevented tau spreading from the CeA to the LC in *PS19* mice. Together, our results suggest that the *Adrb1-A187V* mutation promotes REM sleep, decreases tau spreading, and attenuates tau pathology in *PS19* mice.

## Results

### The *Adrb1-A187V* Mutation Attenuates Tau Accumulation in *PS19* Mice.

**Fig. 1.**
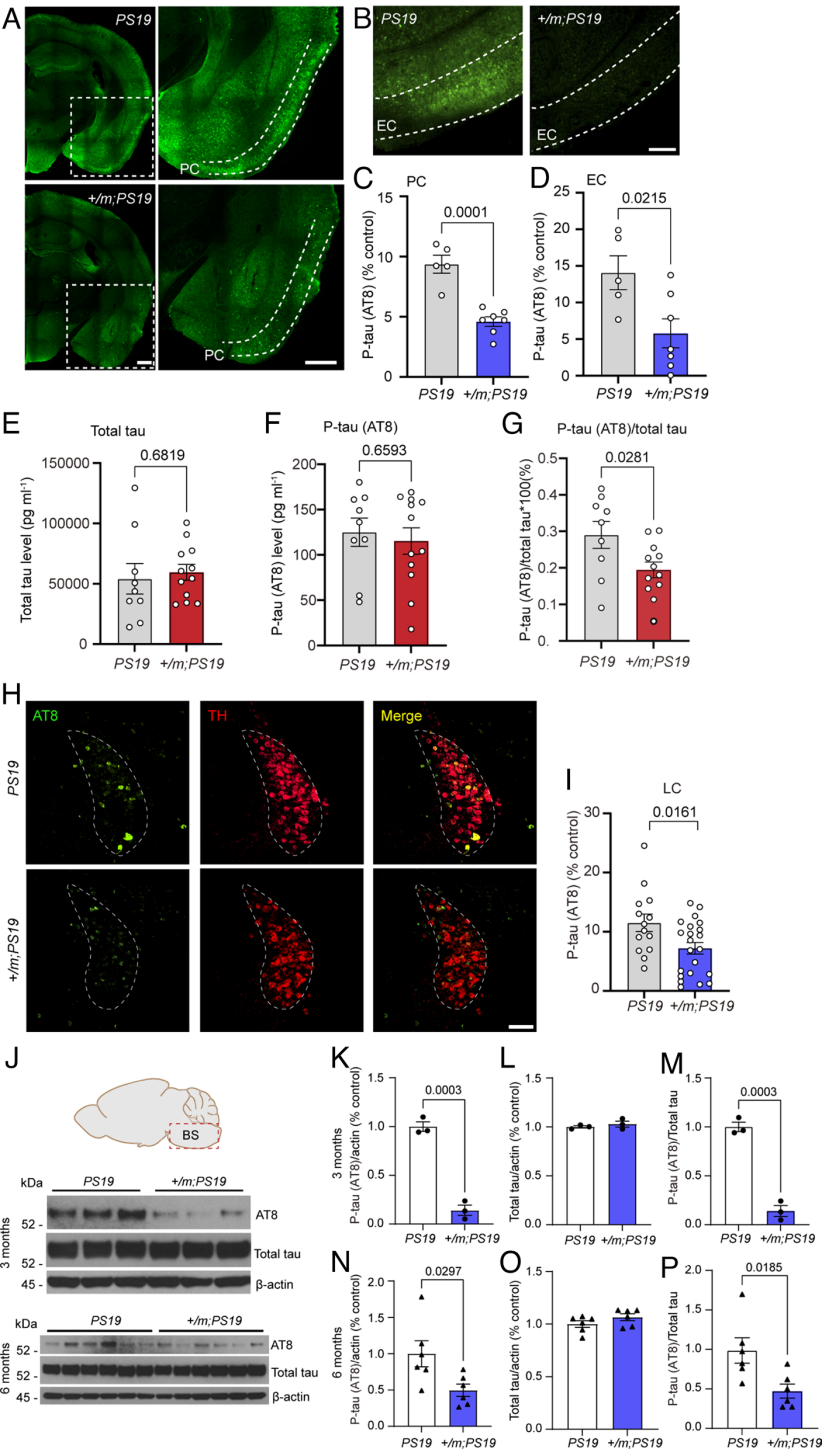
The *Adrb1-A187V* mutation ameliorated tau pathology. (*A* and *B*) Tau pathology indicated by AT8 antibody staining in the piriform cortex (PC) (*A*) and entorhinal cortex (EC) (*B*) of 8-mo-old *PS19* mice and *Adrb1-A187V;PS19* (designated as *+/m;PS19*) mice. (Scale bars, 500 μm.) (*C* and *D*) Area covered by P-tau staining in the PC (*C*) and EC (*D*). Data are means ± SEM; *P* values represent a Student’s two-tailed unpaired *t* test (*C* and *D*). *n* = 5 or *n* = 7 mice per group. (*E*–*G*) ELISA for total tau level (*E*), p-tau (AT8) level (*F*), and the ratio of p-tau (AT8)/total tau (*G*) in the plasma of 8-mo-old mice. *n* = 9 or *n* = 12 mice per group. Data are means ± SEM; *P* values represent a Student’s two-tailed unpaired *t* test (*E*–*G*). (*H*) Tau pathology in the LC of 8-mo-old *PS19* mice and *Adrb1-A187V;PS19* mice. (Scale bar, 100 μm.) (*I*) P-tau (AT8) staining covered area in the LC. *n* = 14 or *n* = 22 mice per group. (*J*) Western blot analysis for p-tau (AT8) and total tau levels in the brainstem (BS) of 3- and 6-mo-old *PS19* and *Adrb1-A187V;PS19* mice. Data are means ± SEM; *P* values represent a Student’s two-tailed unpaired *t* test. (*K–M*) Relative levels of p-tau (AT8) (*K*) and total tau (*L*) and the ratio of p-tau (AT8)/total tau (*M*) in 3-mo-old *PS19* mice and *Adrb1-A187V;PS19* mice for quantification of (*J*). *n* = 3 mice per group. Data are means ± SEM; *P* values represent a Student’s two-tailed unpaired *t* test (*K* and *M*). (*N–P*) The relative levels of p-tau (AT8) (*N*) and total tau (*O*) and the ratio of p-tau (AT8)/total tau (*P*) in 6-mo-old *PS19* mice and *Adrb1-A187V;PS19* mice for quantification of (*J*). *n* = 6 mice per group. Data are means ± SEM; *P* values represent a Student’s two-tailed unpaired *t* test (*N* and *P*).

To determine the effect of the *Adrb1-A187V* mutation on the accumulation of tau pathology, we crossed heterozygous *Adrb1-A187V* mice (*Adrb1+/m*) with *PS19* tau mice expressing 1N4R human tau with a P301S mutation that causes a form of frontotemporal dementia (27) (*Adrb1+/m;PS19*). Heterozygous *Adrb1-A187V* mice were used in all studies to mimic the human mutation carriers who have one mutant allele and one normal allele (16). We used the AT8 antibody to detect p-tau (S202/T205) and found significantly less pathological tau in the piriform cortex (PC) and entorhinal cortex (EC) in 8-mo-old *Adrb1-A187V;PS19* mice vs. *PS19* control mice (Fig. 1 *A*–*D*). However, we did not find any difference in p-tau in the hippocampus of *Adrb1-A187V;PS19* vs. *PS19* mice (*SI Appendix*, Fig. S1). Because phosphorylated tau is a blood-based biomarker for detecting tauopathy, we collected plasma and analyzed total tau and phosphor T181 tau (p-tau) by quantitative enzyme-linked immunosorbent assay (ELISA). Although no significant differences in total tau and p-tau in plasma were detected between *PS19* and *Adrb1-A187V;PS19* mice, the ratio of p-tau/total tau was dramatically reduced in the *Adrb1-A187V;PS19* mice (Fig. 1 *E*–*G*). These results suggest that the *Adrb1* mutation may protect these mice from tau-mediated pathology.

### A Sleep–Wake Regulating Center Accumulates Less Tau in *Adrb1-A187V;PS19* Mice.

The LC is a small nucleus located in the brainstem that has been shown to play a role in some aspects of sleep regulation (20–22). Additionally, postmortem studies have demonstrated that the LC is one of the earliest sites of tau pathology in AD (28, 29). We thus tested whether the LC of the *Adrb1-A187V;PS19* mice is protected from tau pathology by measuring the amount of p-tau staining in the LC of *Adrb1-A187V;PS19* and *PS19* mice. We found that the total area of tau pathology revealed by p-tau staining in *Adrb1-A187V;PS19* mice was significantly less than that found in *PS19* mice (Fig. 1 *H* and *I*). We further analyzed tau protein levels in protein lysates of brainstem at different ages using western blotting. As early as 3 mo of age and prior to the appearance of overt tau pathology, *Adrb1-A187V;PS19* mice showed a robust decrease in p-tau (S202/T205) levels vs. *PS19* mice, while the total tau level was comparable (Fig. 1 *J*–*L*). This result indicates that the lower p-tau level in *Adrb1-A187V;PS19* mice was not due to differences in tau synthesis. At 6 mo of age, with the presence of tau aggregates, *Adrb1-A187V;PS19* mice still had significantly lower p-tau levels than *PS19* mice, while total tau levels remained similar for both genotypes (Fig. 1 *J*, *N*, and *O*). The ratio of p-tau to total tau was significantly lower in *Adrb1-A187V;PS19* vs. *PS19* mice at both 3 and 6 mo of age (Fig. 1 *M* and *P*). Taken together, these results demonstrate that the *Adrb1-A187V* mutation has a potential role in suppressing tau accumulation in the LC, possibly long before the onset of tau pathology.

### Neuronal Transmission Pathways Are Altered in the Cortex of *Adrb1-A187V;PS19* Mutant Mice.

**Fig. 2.**
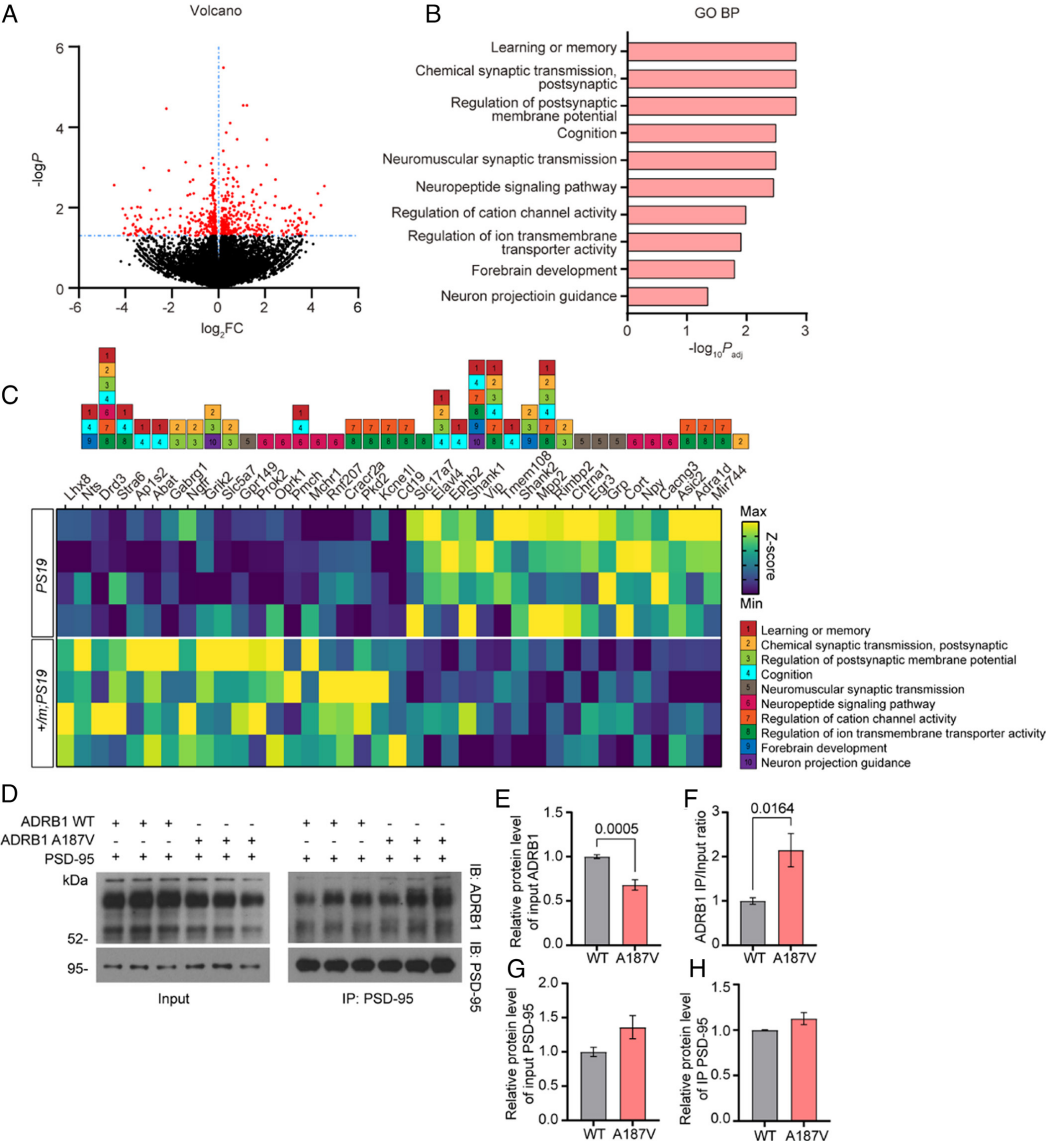
The *Adrb1-A187V* mutation altered neural transmission pathways. (*A*) Volcano plot of DEGs in *Adrb1-A187V;PS19* vs. *PS19* mice. The genes with significantly different expression are shown as red dots (FC > 1.0, *P* < 0.05). (*B*) GO analysis revealed enriched biological processes among the DEGs. (*C*) Heat map of the most differentially expressed genes (curated from the DEGs) among four independent biological replicates. *n* = 4 mice per group. (*D*) Western blots of co-IP results for mutant or WT ADRB1 with PSD-95 antibody. (*E*) The level of mutant ADRB1 was decreased compared to WT ADRB1. (*F*) The ratio of ADRB1 in the IP to input fraction was increased by the ADRB1 mutation. *n* = 9 biological replicates. Data are means ± SEM; *P* values represent a Student’s two-tailed unpaired *t* test with Welch’s correction (*E* and *F*). (*G* and *H*) No significant change difference in PSD-95 levels in either the input (*G*) or IP (*H*) fraction. *n* = 3 biological replicates.

To investigate the molecular mechanism of this protective effect, we compared the gene expression profiles in the cortexes of 8-mo-old *Adrb1-A187V;PS19* and *PS19* mice using bulk RNA-sequencing (RNA-seq) analysis. This analysis identified 555 genes that were differentially expressed, including 284 with increased expression and 271 with decreased expression in *Adrb1-A187V;PS19* vs. *PS19* mice (*P *< 0.05). Genes with altered expression had a distinct transcriptional signature. Gene ontology (GO) enrichment analysis of the differentially expressed genes (DEGs) revealed that the significantly enriched biological pathways for genes with increased expression were mainly involved in learning and memory and neural circuitry-related processes (e.g., synaptic transmission, postsynaptic membrane potential, ion transmembrane transporter activity regulation, and neuropeptide signaling pathways) (Fig. 2 *A* and *B*). Interestingly, we have previously demonstrated that mutant ADRB1 neurons have enhanced excitability and synaptic transmission (16). The heatmap plot confirmed the individual genes implicated in the enriched pathways for the *Adrb1-A187V;PS19* mice (Fig. 2*C*).

β_1_AR was reported to potently regulate memory formation and synaptic plasticity in the mammalian brain by interacting with postsynaptic density protein 95 (PSD-95, a scaffold protein in the post synapses) (30, 31). Therefore, we next examined the interaction between ADRB1 and PSD-95. We first transfected SH-SY5Y cells with wild-type (WT) ADRB1 and/or PSD-95 plasmids to test the colocalization of ADRB1 and PSD-95 in cell culture after treatment with retinoic acid (RA). We observed clear colocalization of ADRB1 with PSD-95 on the membrane of SH-SY5Y cells both 24 and 48 h after transfection (*SI Appendix*, Fig. S2 *A*, *Top* and *Middle*). This finding is consistent with a previous report of such an interaction (30). No colocalization was found in the cells transfected with only ADRB1 or PSD-95 plasmid (*SI Appendix,* Fig. S2 *A*, *Bottom*). We next performed a coimmunoprecipitation (co-IP) assay to confirm the interaction between ADRB1 and PSD-95 by coexpressing WT or mutant ADRB1 (A187V) and PSD-95 in SH-SY5Y cells. Mutant ADRB1 protein levels in the input fraction were significantly decreased compared to WT ADRB1 (Fig. 2 *D* and *E*), consistent with our previous finding of decreased stability of mutant ADRB1 (16). Notably, PSD-95 pulled down more mutant ADRB1 protein than WT control (Fig. 2 *D*, *F*, *G*, and *H*), indicating that the mutation of ADRB1 markedly enhances its interaction with PSD-95. Moreover, WT ADRB1 was clearly present in the PSD-95 pull-down fraction but was absent in both the IgG pull-down fraction (*SI Appendix,* Fig. S2 *B*–*E*) and in cells without PSD-95 coexpression (*SI Appendix,* Fig. S2 *F* and *G*), confirming the specificity of the interaction between ADRB1 and PSD-95. Together, these results suggest that the increased binding of ADRB1 with PSD-95 may contribute to the enhanced synaptic transmission of mutant ADRB1 neurons.

### The LC Receives Projections from CeA^ADRB1^ Neurons.

**Fig. 3.**
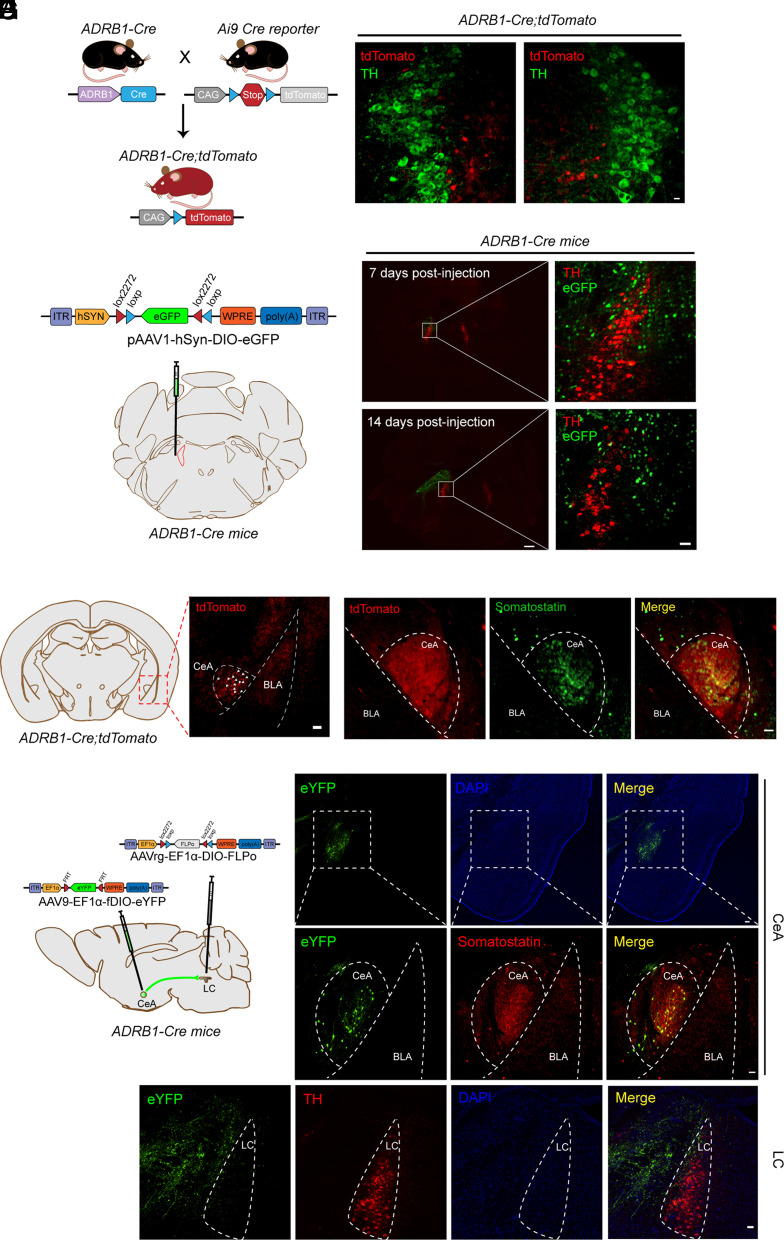
The LC receives projections from CeA^ADRB1^ neurons. (*A*) Schematic of strategy for creating the *ADRB1-Cre;tdTomato* mice. (*B*) Little or no *ADRB1* (tdTomato) signal was detected in the LC (TH). (Scale bar, 50 μm.) (*C*) AAV expression of double inverted ORF (DIO) cassette of eGFP in the region adjacent to the LC of *ADRB1-Cre* mice. (*D*) Expression pattern of ADRB1 (eGFP) 7 and 14 d postinjection of the AAV. [Scale bars, 500 μm (*Left*) and 50 μm (*Right*).] (*E*) ADRB1^+^ (tdTomato) neurons were detected in the CeA. White arrows indicate positive signals. (Scale bar, 50 μm.) (*F*) Costaining of ADRB1^+^ (tdTomato) cells with somatostatin in the CeA. (Scale bar, 50 μm.) (*G*) Schematic of the intersection strategy for determining monosynaptic connections between the LC and CeA^ADRB1^ neurons. (*H*) Representative images showing the robust expression pattern of eYFP in the CeA for the projection-based targeting schematized in (*G*). [Scale bars, 100 μm (*Top*) and 50 μm (*Bottom*).] (*I*) Axon terminals from the CeA^ADRB1^ neurons to the peri-LC region. (Scale bar, 50 μm.)

Since we found significantly less tau aggregation in the sleep center LC (Fig. 1) and the *Adrb1-A187V* mutation modulates sleep features (16), we set out to explore the mechanism by which *Adrb1-A187V* impacts tau aggregation. We first determined the expression of *Adrb1* in the LC and its immediate surrounding areas using two distinct approaches. First, we crossed *ADRB1-Cre* transgenic mice with *Ai9 Cre* tdTomato reporter mice (Fig. 3*A*) and used these mice to examine the colocalization of tdTomato and a marker of the LC (tyrosine hydroxylase, TH). No colocalization was found, indicating that ADRB1 is not expressed in LC neurons (Fig. 3*B*). We next injected adeno-associated virus (AAV) carrying loxP sites flanking enhanced green fluorescent protein (eGFP) into the brain region adjacent to the LC in *ADRB1-Cre* mice (Fig. 3*C*) and examined eGFP expression 7 and 14 d postinjection. Consistent with the genetic results, we observed no colocalization of eGFP with TH-expressing neurons postinjection. Most of the eGFP-positive neurons were in the peri-LC zone (Fig. 3*D*). These results indicate that LC neurons do not express ADRB1.

We next set out to determine whether the mutant *Adrb1* affects the tau pathology in the LC via projections to LC neurons. We first screened the brain regions previously reported (32–34) to send projections to the LC by detecting *ADRB1*-expressing neurons in the *ADRB1-Cre;Ai9* mice. We found robust tdTomato positive signals (ADRB1^+^) in the CeA neurons, but little or no obvious signals in other candidate areas (Fig. 3*E* and *SI Appendix,* Fig. S3 *A*–*D*). Costaining for ADRB1 and the CeA marker somatostatin confirmed that most of the CeA neurons were ADRB1^+^ (Fig. 3*F*).

The CeA is a complex structure containing heterogeneous neuronal populations. It projects to various nuclei in addition to the LC, including the periaqueductal gray, nucleus tractus solitarius, and hypothalamus (35–37). To determine whether ADRB1^+^ cells in the CeA (CeA^ADRB1^ neurons) send projections to the LC, we used retrograde tracing. AAVrg is an AAV variant that can be taken up and retrogradely transported by the axonal terminals of projection neurons (38). For this experiment, we injected the AAVrg construct (AAVrg-cDIO-Flp) into the LC and another AAV construct (AAV9-fDIO-eYFP) into the CeA of *ADRB1-Cre* transgenic mice (Fig. 3*G*). Then, only CeA neurons that both expressed Cre (ADRB1^+^) and were transduced by AAVrg (LC projecting) could activate Flp-dependent eYFP expression. Indeed, we found strong eYFP signals in the CeA, where we also observed positive staining for somatostatin (Fig. 3*H*). In addition, the eYFP-positive projection fibers were abundant in the peri-LC (Fig. 3*I*) with some terminating within the LC area, indicating that the LC receives dense terminal innervations from CeA^ADRB1^ neurons. Together, these results demonstrate that ADRB1^+^ neurons in the CeA project to the LC.

### The *Adrb1-A187V* Mutation Attenuates tau Spreading from the CeA to LC.

**Fig. 4.**
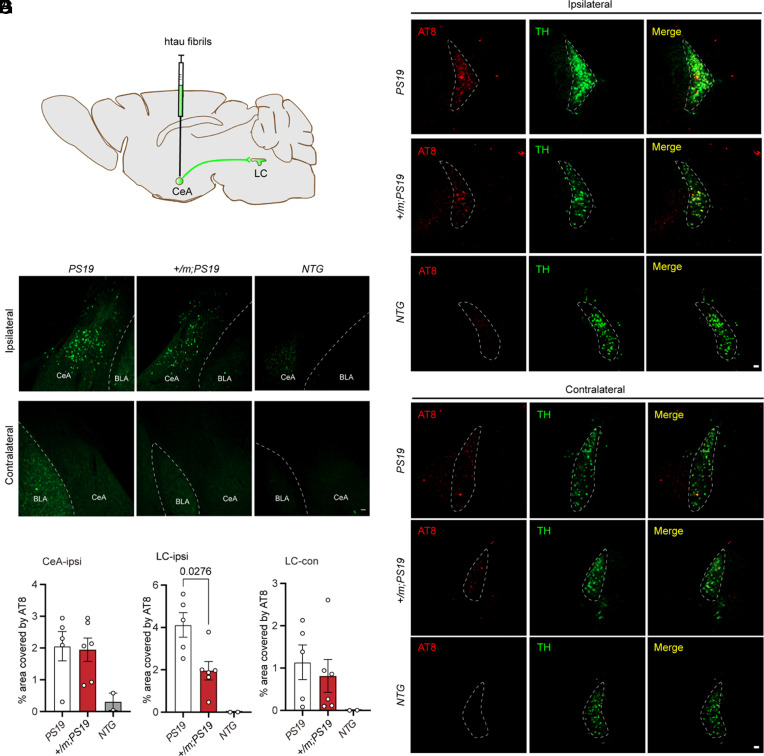
The *Adrb1-A187V* mutation mitigated tau spreading from the CeA to the LC. (*A*) Schematic drawings of tau fibril injection into the CeA. (*B*) *Top*, ipsilateral CeA p-tau staining in *PS19Adrb1-A187V;PS19* and nontransgenic (*NTG*) mice with unilateral CeA tau fibril injection; bottom, contralateral CeA AT8 p-tau staining. (Scale bar, 50 μm.) (*C*) Percentage area covered by AT8 staining in the ipsilateral CeA of *PS19Adrb1-A187V;PS19,* and *NTG* mice for (*B*). From *Left* to *Right*, *n* = 5, *n* = 6, and *n* = 2 mice per group. (*D* and *E*) AT8 staining in the ipsilateral (*D*) and contralateral (*E*) LC of CeA-seeded *PS19Adrb1-A187V;PS19,* and *NTG* mice. (Scale bars, 50 μm.) (*F* and *G*) p-Tau covered area in % in the ipsilateral (*F*) for the quantification of (*D*) and the contralateral LC (*G*) for the quantification of (*E*). From *Left* to *Right*, *n* = 5, *n* = 6, and *n* = 2 mice per group. Data are means ± SEM; *P* values represent a one-way ANOVA with Sidak’s multiple comparison’s test (*F*).

Since tau protein spreads transsynaptically in human and mouse neurons (39, 40) and chronic SD increases the spread of tau pathology to a synaptically connected brain region (14), we tested whether the *Adrb1* mutation in *PS19* mice can lead to less tau spreading to the LC from the CeA. Recombinant human tau fibrils were injected unilaterally into the CeA of 2-mo-old *Adrb1-A187V;PS19* and *PS19* mice, prior to the presence of tau pathology (Fig. 4*A* and *SI Appendix,* Fig. S4). Thirty days after injection, tau pathology was primarily restricted to the ipsilateral CeA in both *Adrb1-A187V;PS19* and *PS19* mice, as assessed by p-tau staining (Fig. 4 *B*, *Top*). No obvious tau aggregates accumulated either on the contralateral side or in the nontransgenic mice (*NTG*) (Fig. 4 *B*, *Bottom*). Tau pathology in the CeA of *Adrb1-A187V;PS19* mice was comparable to that of *PS19* mice (Fig. 4*C*), suggesting that the *Adrb1-A187V* mutation does not affect tau seeding in the CeA. Intriguingly, we saw a significant decrease in p-tau staining on the ipsilateral side of the LC in the *Adrb1-A187V;PS19* mice compared to the control mice (Fig. 4 *D* and *F*). No obvious staining was detected on the contralateral side (Fig. 4 *E* and *G*). These results indicate that the *Adrb1-A187V* mutation can significantly reduce the propagation of tau in the CeA to the downstream LC.

### The *Adrb1-A187V* Mutation Mitigates the REM Sleep Reduction in *PS19* Mice.

**Fig. 5.**
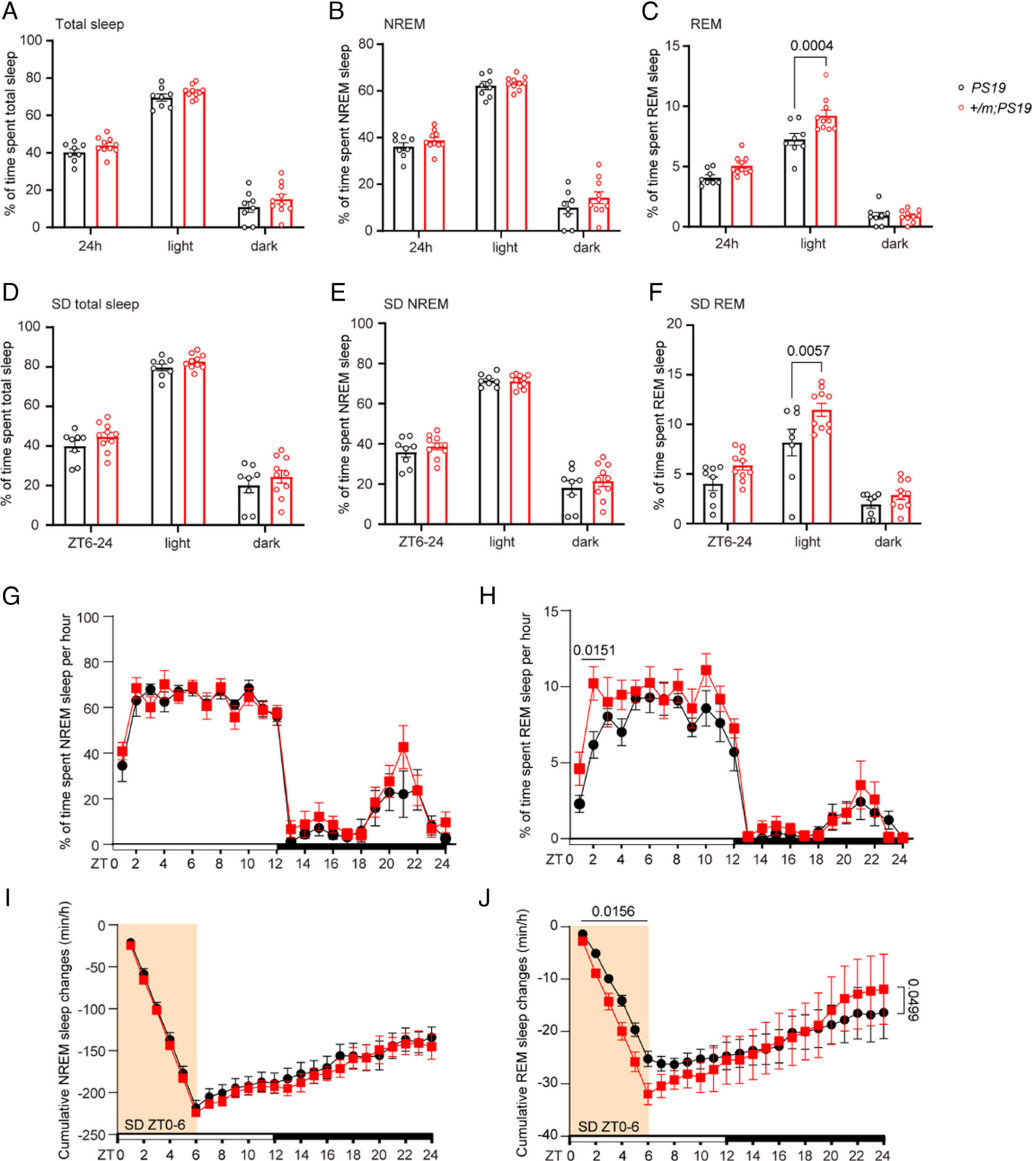
Sleep of the *Adrb1-A187V;PS19* mice compared to *PS19* mice. (*A*–*C*) EEG/EMG analysis of percent total sleep (*A*), percent NREM sleep (*B*), and percent REM sleep (*C*) under normal conditions. *n* = 8 or *n* = 10 mice per group. Data are means ± SEM; *P* values represent a two-way ANOVA with Sidak’s multiple comparisons test (*C*). (*D*–*F*) EEG/EMG analysis of percent total sleep (*D*), percent NREM sleep (*E*), and percent REM sleep (*F*) after manual sleep deprivation (SD) for 6 h. *n* = 8 or *n* = 10 mice per group. Data are means ± SEM; *P* values represent a two-way ANOVA with Sidak’s multiple comparisons test (*F*). (*G* and *H*) EEG/EMG analysis of percent NREM (*G*) and REM (*H*) sleep per hour. *n* = 7 to 8 mice per group. Data are means ± SEM; *P* values represent a two-way ANOVA with Sidak’s multiple comparisons test (*H*). (*I* and *J*) Cumulative changes in NREM (*I*) and REM (*J*) sleep time during SD and recovery periods compared to the baseline days**.** *n* = 7 to 8 mice per group. Simple linear regression (*J*).

Previously, *PS19* mice were reported to have a noticeable reduction in REM sleep duration at 9 mo of age (26). We therefore examined whether the *Adrb1* mutation alters sleep architecture in *PS19* mice using continuous electroencephalography (EEG)/electromyography (EMG) monitoring for 48 h. We observed no significant differences in total sleep time and nonrapid eye movement (NREM) sleep time in *Adrb1-A187V;PS19* vs. *PS19* mice (Fig. 5 *A*, *B*, and *G*). However, the REM sleep time was significantly increased in the *Adrb1-A187V;PS19* mice compared to the *PS19* mice during the light phase (Fig. 5 *C* and *H*), suggesting that the *Adrb1-A187V* mutation can help at least partly restore the loss of REM sleep seen in *PS19* mice. To further dissect the effects of the *Adrb1* mutation on sleep, we subjected mice to 6 h of SD before examining their sleep–wake features. We found that both *Adrb1-A187V;PS19* and *PS19* mice had similar NREM sleep recovery after SD (Fig. 5 *D* and *E*). However, the *Adrb1-A187V;PS19* mice showed higher recovery of REM sleep compared with *PS19* mice (Fig. 5*F*). We next examined the kinetics of sleep loss and recovery by calculating cumulative changes in the amounts of NREM and REM sleep compared to those on baseline days. During the 6 h of SD, *Adrb1-A187V;PS19* mice and *PS19* mice accumulated similar NREM sleep deficits; however, *Adrb1-A187V;PS19* mice had a greater REM sleep deficit than *PS19* mice (Fig. 5 *I* and *J*; ZT0-6), which is likely due to the higher amount of REM sleep in *Adrb1-A187V;PS19* mice on baseline days. During the subsequent 18-h recovery period, both groups recovered NREM sleep at the same rate (Fig. 5*I*; ZT6-24). Remarkably, *Adrb1-A187V;PS19* mice exhibited a faster rate of REM sleep recovery than *PS19* mice (Fig. 5*J*; ZT6-24), indicating that *Adrb1-A187V;PS19* mice had stronger REM sleep rebound than *PS19* mice. These results suggest that the *Adrb1* mutation can mitigate the reduction of REM sleep in *PS19* mice.

### Chemogenetic Manipulation of CeA^ADRB1^ Neurons Increases REM Sleep.

**Fig. 6.**
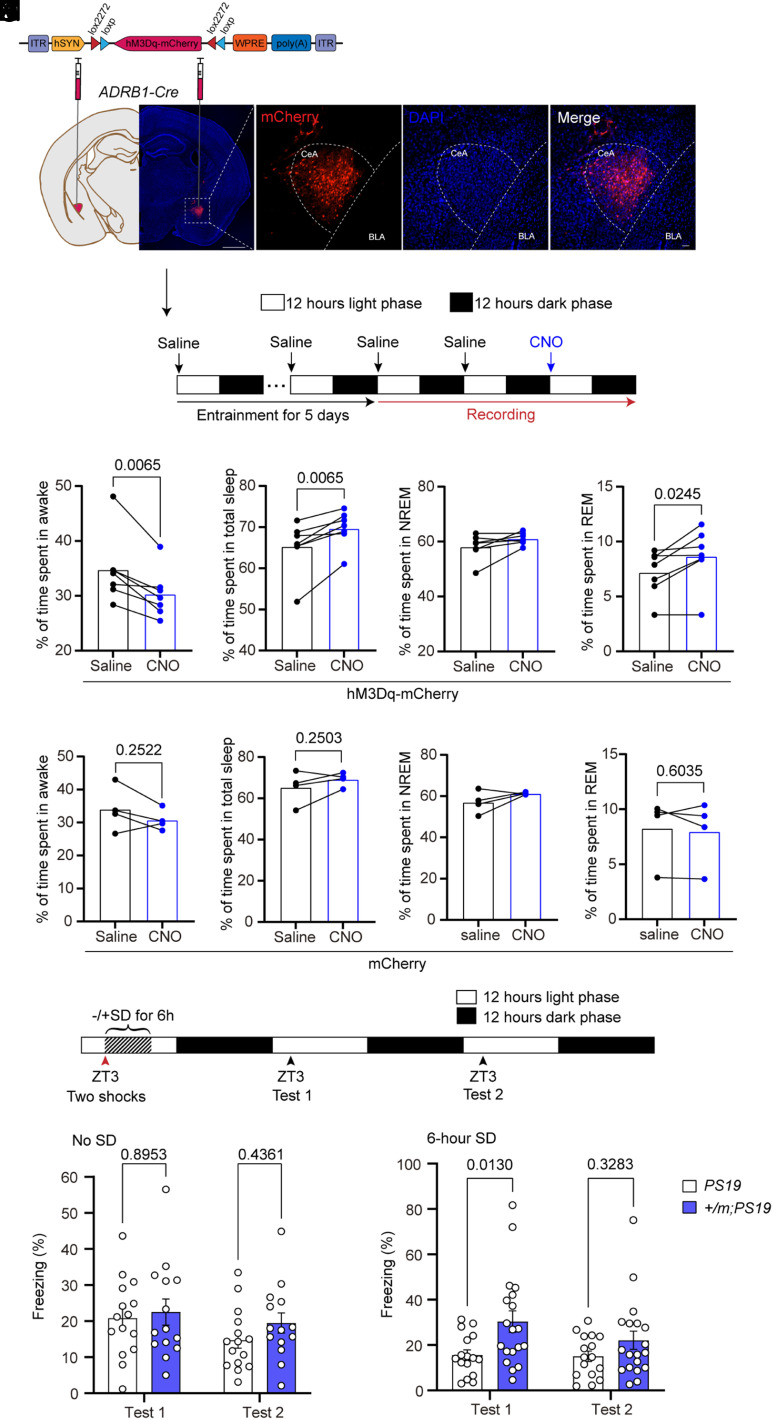
Chemogenetic activation of CeA^ADRB1^ neurons reduced wake and increased REM sleep. (*A*) hM3Dq-mCherry or mCherry expression in ADRB1^+^ cells of the CeA was achieved by injection of AAV-hSYN-DIO-hM3Dq-mCherry or AAV-hSYN-DIO-mCherry bilaterally into the CeA of 2 to 3-mo-old *ADRB1-Cre* mice. [Scale bars, 1 mm (*Left*) and 50 μm (*Right*).] (*B*) Experimental design. (*C*–*F*) Durations of awake time (*C*), total sleep (*D*), NREM sleep (*E*), and REM sleep (*F*) in mice infused with hM3Dq-mCherry during the first 6 h after 5 mg/kg CNO or saline administration. *n* = 7 mice per group. Data are means ± SEM; *P* values represent a paired two-tailed *t* test (*C*, *D*, and *F*). (*G*–*J*) Durations of awake time (*G*), total sleep (*H*), NREM sleep (*I*), and REM sleep (*J*) of mice infused with mCherry during the first 6 h after 5mg/kg CNO or saline administration. Data are means ± SEM; *P* values represent a paired two-tailed *t* test (*G*, *H*, and *J*). (*K*) Schematic representation of the timeline of contextual fear conditioning experiments with or without 6 h of SD. (*L*) Percent freezing time of 6 to 8-mo-old mice without SD. *n* = 15 or *n* = 14 mice per group. (*M*) Percent freezing time after SD. *n* = 16 or *n* = 19 mice per group. Data are means ± SEM; *P* values represent a two-way ANOVA with Sidak’s multiple comparisons test (*L* and *M*).

Although the LC is not active during REM sleep, the amygdala is active during REM sleep (41). Based on our finding that the Adrb1 mutation reduces tau spreading from the CeA to LC (a known sleep regulation center) and mitigates REM sleep reduction in *PS19* mice (Fig. 5), we investigated whether CeA^ADRB1^ neurons play a role in sleep–wake regulation. We applied DREADDS (designer receptors exclusively activated by designer drugs) to specifically activate CeA^ADRB1^ neurons in *ADRB1-Cre* mice and confirmed that hM3Dq-mCherry or mCherry expression was mainly restricted to the CeA (Fig. 6*A*). Administration of clozapine-N-oxide (CNO) to the hM3Dq-mCherry mice increased c-FOS expression in CeA, confirming the activation of these neurons (*SI Appendix*, Fig. S5). We found that CNO administration led to a marked reduction in the amount of time spent awake during the first 6 h after treatment (Fig. 6 *B* and *C*). Notably, concomitant increases in the durations of total sleep and REM sleep were observed with CNO treatment (Fig. 6 *D* and *F*). However, no significant change in the NREM sleep time was observed (Fig. 6*E*). To test whether the effects of CNO were specific to hM3Dq and not due to off-target effects, we also injected the virus carrying mCherry into *ADRB1-Cre* mice. We found that systemic administration of CNO did not alter sleep–wake behavior in these mice (Fig. 6 *G*–*J*), suggesting that neither CNO nor its metabolite clozapine influences the sleep or wake time in the absence of hM3Dq expression. These results indicate that the hM3Dq-mediated activation of CeA^ADRB1^ neurons can reduce wakefulness and enhance REM sleep. We have previously shown that mutant ADRB1 neurons have higher activity than nonmutant ADRB1 neurons (16). Altogether, these results suggest that mutant ADRB1 neurons in the CeA may contribute to the restoration of REM sleep in *PS19* mice. Further investigation is needed to determine whether additional brain areas are also involved in this REM restoration effect.

### The *Adrb1-A187V* Mutation Protects *PS19* Mice from Sleep Loss-Induced Memory Deterioration.

AD patients suffer from cognitive decline including memory deterioration, and one of the FNSS mutations was previously shown to protect against memory loss caused by reduced sleep (17). We thus set out to test whether the *Adrb1* mutation also modulates memory in *PS19* mice. We subjected mice to the contextual fear conditioning test, which is sensitive to sleep conditions. *Adrb1-A187V;PS19* and *PS19* mice underwent training and were then either subjected to acute SD (experimental group) or allowed to sleep ad libitum (control group). The mice were then tested for the percentage of freezing time at the same time point on the following 2 d (Fig. 6*K*). The *Adrb1-A187V;PS19* mice showed comparable freezing times to *PS19* mice under baseline conditions (Fig. 6*L*), but the double-mutant mice had a dramatically higher percentage of freezing time than the *PS19* mice on the first testing day after 6 h of acute SD (Fig. 6*M*). In addition, the freezing times of *Adrb1-A187V;PS19* and *PS19* mice did not differ and were comparable to baseline measures on the 2nd day of testing, suggesting that the memory extinction process remained intact. Together, these findings demonstrate that the *Adrb1-A187V;PS19* mice are more resistant to SD-induced memory loss than *PS19* mice.

## Discussion

Sleep homeostasis is vital for human health and well-being, and sleep helps to clean out the metabolic waste that builds up in the brain during wakefulness (42). Interestingly, protein aggregation in the brain is a hallmark of many neurodegenerative diseases, and poor sleep quality is common among patients with these conditions. We originally tested the hypothesis that quality sleep can alleviate AD pathology, including Aβ plaque and tau tangle accumulation, with mouse models of AD and FNSS, and found that two FNSS mutations can protect *PS19* mice from tauopathy (19), though the underlying mechanism remains unclear. To further confirm this finding and gain more insight into the role of FNSS mutations in tauopathy, we crossed another FNSS mouse model, *Adrb1-A187V* mice, with *PS19* mice to investigate the effects of this mutation on tau pathology. Consistent with our previous findings, we found that *Adrb1-A187V;PS19* mice also had fewer tau aggregates in the cortex and less p-tau in plasma than *PS19* mice, indicating that the *Adrb1* FNSS mutation also has a protective function against accumulation of tau pathology.

Gene expression analysis of the cortex revealed that the *Adrb1-A187V* mutation altered pathways associated with neuronal circuits (synaptic transmission, postsynaptic potential, and neuron projection guidance) in *PS19* mice. Previous studies reported that *Adrb1* potently regulates memory formation and synaptic plasticity (31, 43, 44), and ADRB1 likely functions through interactions with PSD-95, which facilitates complex formation with N-methyl-d-aspartate receptors for modulating synaptic transmission (30). We have also demonstrated enhanced excitability and increased synaptic transmission in ADRB1 mutant neurons (16). Here, we found that mutant ADRB1 has a stronger interaction with PSD-95 than WT ADRB1, supporting the notion that the *Adrb1-A187V* mutation potentially strengthens synaptic plasticity and memory through its increased binding with PSD-95. Consistent with these observations, pharmacological activation of β-ARs was shown to reverse memory impairment in sleep-deficient rats (45). Interestingly, we found that the *Adrb1-A187V* mutation can significantly increase the resilience of sleep-deprived *PS19* mice to memory loss.

*PS19* mice show reduced REM sleep at 9 mo of age compared with WT controls (26), and we found that the *Adrb1-A187V* mutation helped restore the decreased REM sleep in *PS19* mice. However, no change in NREM sleep was observed between *Adrb1-A187V;PS19* and *PS19* mice, indicating that the *Adrb1-A187V* mutation specifically alters REM sleep. β-ARs in the brain have been shown to be associated with REM sleep in rats (46). We have also demonstrated that the ADRB1^+^ neurons in two different areas of brainstem (dorsal pons and peritegmental reticular nucleus) are active during REM sleep and that the activity of ADRB1^+^ neurons begins to increase before NREM-to-REM transitions (16, 47). Collectively, the evidence indicates a role for ADRB1 in REM sleep regulation. Furthermore, after 6 h of SD, we observed that *Adrb1-A187V;PS19* mice had faster recovery of REM sleep than *PS19* mice, supporting the conclusion that *Adrb1-A187V;PS19* mice are more resistant than *PS19* mice to the negative effects of sleep deficits.

Noradrenergic dysfunction is common in AD-related pathology (48–50). The LC is the main source of NE in the brain with widespread efferent projections and is known to be involved in sleep–wake regulation (51). It has been reported that the integrity of the LC is associated with tau burden and memory loss in AD patients (52). Remarkably, we found less p-tau protein and tau accumulation in the LC of 8-mo-old *Adrb1-A187V;PS19* vs. *PS19* mice. To further investigate how mutant *Adrb1* regulates tau pathology in the LC, we searched for ADRB1-positive brain regions sending projections to the LC. We found that among the many areas sending efferents into the LC, the CeA was the only area with ADRB1^+^ neurons, suggesting that ADRB1 likely functions through a CeA–LC circuit to modulate tau pathology in the LC. In addition, the *Adrb1-A187V;PS19* mice also showed significantly less tau spreading from the CeA to the LC compared with *PS19* mice. Overall, our findings demonstrate that the *Adrb1-A187V* mutation has a protective effect against tau pathology, in part, by ameliorating tau accumulation in the LC and attenuating tau spreading from the CeA to the LC.

Chemogenetic stimulation of ADRB1^+^ neuronal activity in the CeA increased REM sleep in *PS19* mice with decreased wakefulness, and mutant ADRB1 neurons were more active, which would mimic the activating effect by experimental stimulation. Nonetheless, it remains to be determined whether the activity of mutant ADRB1 neurons in the CeA is directly and/or the sole contributor to the overall REM sleep increase seen in *Adrb1-A187V;PS19* mice. Further research is also needed to reveal whether the enhanced REM sleep in these mice plays a role in mitigating the tau pathology.

## Methods

### Nomenclature.

*“Adrb1”* represents the mouse gene, and “*ADRB1”* represents the human gene. The corresponding protein is denoted by “Adrb1” for the mouse receptor and “ADRB1” for the human receptor.

### Animals.

All animal experiments were performed at University of California, San Francisco (UCSF), and all experimental protocols were approved by the UCSF Institutional Animal Care and Use Committee following the NIH’s *Guide for the Care and Use of Laboratory Animals.*

*Adrb1-A187V* knock-in mice were generated by our group via CRISPR/Cas9 (16). Briefly, the sgRNA targeting *Adrb1* gene was transcribed in vitro, then brought together with Cas9 protein and donor oligonucleotide, and microinjected into the pronucleus of fertilized C57BL/6J zygotes. The injected zygotes were implanted into the oviducts of pseudopregnant CD1 female mice to create positive knock-in founders. The mice were backcrossed with C57BL/6J to dilute out potential off-target affects. The *PS19* transgenic mouse strain was kindly provided by Li Gan. The mice express the human 1N4R tau isoform carrying P301S mutation, driven by the mouse prion protein promoter, and were maintained by backcrossing to C57B/6J mice. Double transgenic mice were generated by crossing the *Adrb1-A187V* mice with the *PS19* line. *ADRB1-Cre* BAC transgenic mice were generated in our lab as previously described (16). We modified the human BAC clone CTD 2337B3 with the entire *ADRB1* gene in a 150-kb genomic insert by replacing the coding sequence of *ADRB1* with that of a Cre recombinase via homologous recombination. The modified BAC DNA was sequence confirmed and injected into C57BL/6J embryos following standard procedures. Ai9 (stock no. 007909) Cre reporter mice were purchased from the Jackson Laboratory. Mice were given ad libitum access to food and water with a 12-h light/12-h dark cycle. Male mice aged 3 mo old and 6 to 8 mo old were used for all experiments unless otherwise noted. Littermates were used for controls.

### Immunofluorescence.

Immunofluorescence staining was performed as previously described (19). Mouse brains were collected after transcardial perfusion with ice-cold 1× phosphate-buffered saline (PBS) and postfixed in 4% paraformaldehyde overnight at 4 ℃. Then, brains were incubated in a 30% sucrose solution for 24 h at 4 ℃ before being embedded in Tissue-Plus™ (Optimal Cutting Temperature compound; Fisher Scientific) and frozen at −80 ℃. Three to five coronal brain sections (40 μm thickness) from each mouse were used for free-floating immunofluorescent staining. Brain sections were incubated with the primary antibodies AT8 (Thermo Scientific, MN1020, 1:500), antisomatostatin antibody (Abcam, ab108456, 1:100), and antityrosine hydroxylase (TH) antibody (Invitrogen, P21962, 1:1,000). Secondary antibodies included goat anti-mouse Alexa Fluor® 488 (Abcam, ab150113, 1:500), goat anti-rabbit Alexa Fluor® 488 (Abcam, ab150077, 1:500), and goat anti-rabbit-Cy3 (Invitrogen, A10520, 1:1,000). Slices were mounted in a mounting medium with DAPI and DABCO™ (EMS). All data obtained were analyzed by investigators blinded to the genotypes of the mice. For immunocytochemistry of cultured SH-SY5Y cells transiently transfected with WT/A187V ADRB1 plasmid and PSD-95 plasmid (gift from Wei-dong Yao, Addgene, 15463), the cells were first fixed with 100% methanol (chilled at −20 °C) at room temperature (RT) for 5 min. Then, the cells were washed with ice-cold 1× PBS three times and stained as described above.

### ELISA.

Blood was collected into commercially available EDTA-treated tubes (BD Vacutainer^®^ blood collection tubes). Samples were spun at 2,000 g for 10 min at 4 ℃ to obtain plasma. ELISAs for human total tau or p-tau in plasma were performed according to the manufacturer’s instructions for human total tau or p-tau ELISA kits (Invitrogen) with brief modifications. For assays of total tau levels, 50 μL plasma was incubated overnight at 4 ℃; for assaying p-tau levels, plasma was diluted 250 times and incubated for 2 h at RT. Absorbance was read at 450 nm using a Synergy H4 Hybrid Multi-Mode Microplate Reader (BioTek), and all measurements and analysis of data obtained were performed by investigators blinded to mouse genotypes.

### Contextual Fear Conditioning.

Mice were handled for at least 1 min per day for 1 wk before the behavioral experiment. The mice were habituated to testing room conditions for 1 h before the test was started (3 h after the onset of light period–ZT3). Each mouse was placed in a test chamber and allowed to explore freely for 3 min followed by two mild (2 s, 0.72 mA) electric foot shocks with a 1-min interval between them. The mouse was left in the chamber for an additional 1 min before being returned to its home cage. Contextual testing was performed 24 and 48 h after training (electric foot shocks), and each mouse was placed in the same chamber for 5 min without foot shocks for measurement of the freezing time. For the measurements with SD, mice were subjected to SD for 6 h immediately following the foot stimulation and then returned to their home cages. Data were acquired using ANY-maze automated tracking software, and the percentage of freezing time to total testing time was recorded.

### Adenoassociated Viral (AAV) Vectors.

pAAV-hSyn-DIO-eGFP (Addgene viral prep # 50457-AAV1; http://n2t.net/addgene:50457; RRID:Addgene_50457), pAAV-hSyn-DIO-hM3D(Gq)-mCherry (Addgene viral prep # 44361-AAV8; http://n2t.net/addgene:44361; RRID:Addgene_44361), and pAAV-hSyn-DIO-mCherry (Addgene viral prep # 50459-AAV8; http://n2t.net/addgene:50459; RRID:Addgene_50459) were gifts from Bryan Roth. pAAV-Ef1a-fDIO-eYFP was a gift from Karl Deisseroth (Addgene viral prep # 55641-AAV9; http://n2t.net/addgene:55641; RRID:Addgene_55641). pAAV-EF1a-DIO-FLPo-WPRE-hGHpA was a gift from Li Zhang (Addgene viral prep # 87306-AAVrg; http://n2t.net/addgene:87306; RRID:Addgene_87306).

### Stereotactic Injection.

Mice were briefly anesthetized with isoflurane and placed in a stereotaxic frame (Kopf). Administration of 1 to 2% isoflurane was used to keep the mice under anesthesia during surgery. Following head fixation and testing for withdrawal response, a longitudinal incision was made along the midline to expose the skull. The dura was punctured using a 23-gauge surgical needle. Virus was injected stereotactically with a glass capillary into the target region at a rate of 100 nL/min using a microinjection syringe pump (UMP3; WPI). The glass capillary was kept at the injection site for an additional 10 min and then slowly withdrawn at 0.01 mm/s. The mice were kept warm on a heating pad and monitored until they fully recovered from anesthesia.

For detecting ADRB1 expression in the LC of *ADRB1-Cre* mice, AAV1-DIO-eGFP was injected adjacent to the LC (anterior–posterior [AP] = −5.45, medial–lateral [ML] = −1.23, dorsal–ventral [DV] = −3.65) in a total volume of 300 nL. Virus expression was analyzed 7 and 14 d postinjection.

For intersectional injection, 300 nL AAVrg-DIO-FLPo was injected in the LC (AP = −5.45, ML = −1.23, DV = −3.65), and 300 nL AAV-fDIO-eYFP was simultaneously injected in the CeA (AP = −1.22; ML = −2.75; DV = −4.5). After recovery, mice were housed in home cages for 14 d before euthanasia.

For DREADD experiments, 300 nL/site AAV8-DIO-hM3Dq-mCherry or AAV8-DIO-mCherry was stereotactically injected bilaterally with a glass capillary into the CeA (AP = −1.22; ML = ±2.75; DV = −4.5). The EEG/EMG electrodes were implanted following the injection of the AAV8-DIO-hM3Dq-mCherry. Expression of hM3Dq in CeA^ADRB1^ neurons was allowed for ~3 wk and histologically assessed in all mice after the experiments. Three weeks after DREADD expression, intraperitoneal saline was administered at the start of the light phase for 5 d to acclimate the mice to the injections. Then, EEG/EMG recordings began. The mice were then treated with saline for 2 more days as a baseline control followed by 5 mg/kg CNO (Sigma-Aldrich) treatment to manipulate neuron activity. Saline and AAV8-DIO-mCherry treatment served as the control.

For tau pathology seeding and spreading, 8 to 9-wk-old *Adrb1-A187V;PS19* mice or *PS19* mice were unilaterally injected with 4 µg preformed fibrils of active human recombinant tau protein carrying a P301S mutation (StressMarq Biosciences Inc., Catalog # SPR-329) in the CeA (AP = −1.22; ML = −2.75; DV = −4.5) in a final volume of 2 µL with an infusion rate of 0.2 µL/min. After 4 wk of recovery, mouse brain was collected for measurement of tau pathology seeding and spreading.

### EEG/EMG Implantation.

For EEG/EMG implantation, one ground stainless steel screw and three screws with insulated wires were soldered to a 6-pin connector EEG/EMG headset (Pinnacle Technologies, Kansas) as previously described (16). Two screws were placed rostral and lateral to the bregma over the frontal bone (AP = +1.00; ML = ±1.25); the left one served as the ground electrode. The other two screws were placed caudal and lateral to the bregma above the parietal bone (AP = −3.0; ML = ±2.5). EMG electrodes were directly inserted into the cervical trapezius muscle. An EEG headset was secured with dental cement, and the skin was stitched. Mice were singly housed to recover for 8 to 10 d before transfer into recording chambers in a 12-h light/12-h dark cycle. Flexible recording cables were attached to the EEG/EMG headset to allow the mice to habituate for 1 wk before recording started.

### EEG/EMG Recording and Analysis.

EEG/EMG signals were amplified with a preamplifier and digitally acquired by the Sirenia software package (Pinnacle Technologies). The signals were recorded for 48 h, and all data were sampled at 500 Hz. EEG/EMG recordings were manually scored in 10-s epochs by investigators blinded to genotype using Sirenia Sleep Pro software. Recordings were scored for wakefulness, NREM sleep, and REM sleep, and artifact epochs were excluded. The percentages of time spent in wake vs. NREM sleep vs. REM sleep were summarized for each group and each condition and averaged across 48 h. NREM sleep was classified as high-amplitude slow-wave EEG signal (delta rhythm, 0.5 to 4 Hz) with no muscle activity on EMG. REM sleep was classified as rapid low-amplitude wave EEG signal (theta rhythm, 4 to 8 Hz) with little or no EMG signal. Other epochs were marked as wake with low-to-moderate amplitude EEG and occurrence of EMG signals. EEG power spectra were computed using a fast Fourier transfer routine. The time course of EEG delta power (0.5 to 4.0 Hz) during NREM sleep was calculated as previously described (16). The values were normalized to the individual mean delta power value scored as NREM sleep during ZT9-ZT12 of the rest period. For the kinetics of sleep loss and recovery analysis, we calculated the cumulative changes in NREM and REM sleep duration. The amounts of NREM and REM sleep/hour on the baseline day were subtracted from those on the SD day. Then, the cumulative deficit was computed across the SD and recovery periods. The sleep loss and gain rates were calculated by linear regression over the SD and recovery periods, respectively.

### Protein Extraction and Western Blotting.

For brain lysate analysis, mouse brainstem was collected and homogenized in radioimmunoprecipitation assay (RIPA) buffer (50 mM Tris pH 8.0, 150 mM NaCl, 1% Nonidet P-40, 0.5% nadeoxycholate, 5 mM EDTA, 0.1% sodium dodecyl sulfate) with protease inhibitor (Sigma-Aldrich), phosphatase inhibitor (Roche), and 1 mM PMSF. Samples were separated on 4 to 12% NuPAGE (Invitrogen) gel with MOPS buffer and then transferred to polyvinylidene difluoride membranes (Millipore). For cell culture lysate analysis, cells were washed with ice-cold 1× PBS and lysed with ice-cold RIPA buffer (500 μL/100-mm dish) in the presence of protease and phosphatase inhibitors. Then, cell lysates were centrifuged at 12,000 rpm for 10 min at 4 ℃ to obtain proteins. Then, PSD-95 antibody or IgG was incubated with the protein extract on a rotator overnight at 4 ℃. The next day, protein G agarose beads (Thermo Scientific, 20398) were added to the mixture, which was then incubated for 2 h at 4 ℃. The immunoprecipitated proteins were eluted by boiling for 10 min in Laemmli buffer and analyzed by western blotting. Primary antibodies used in this study were AT8 (Thermo Scientific, MN1020), HT7 (Thermo Scientific, MN1000), anti-β-actin (Cell Signaling Technology, 4967S), anti-ADRB1 (Abcam, ab3442), and anti-PSD95 (Abcam, ab18258). Membranes were developed using horseradish peroxidase substrate (Millipore) and exposed to X-ray film (Thermo Scientific).

### RNA-seq Analysis.

Mouse cortical tissue was dissected, and total RNA was extracted and sequenced using the Illumina HiSeq 2500 platform (paired-end 150 nt read length, Novogene). RNA-seq resulted in 46.8 million reads/sample, on average. The raw reads of the sequencing data were submitted to NCBI-GEO under accession number *GSE205558*. Clean reads were aligned to the mouse reference genome (GRCm38/mm10) using Hisat2 (version 2.0.5). Quantification of gene expression was analyzed by featureCounts (version 1.5.0-p3). Differential gene expression levels between mutant and WT tissues were analyzed using the DESeq2 R package (version 1.20.0). DEGs were selected with criteria of *P* < 0.05, and fold change > 1. GO enrichment analysis of DEGs was performed using the clusterProfiler R package (version 3.8.1), and GO terms with adjusted *P* values < 0.05 were considered significantly enriched by DEGs.

### Cell Culture and Transfection.

SH-SY5Y neuroblastoma cells (ATCC; CRL-2266) were cultured with Dulbecco’s Modified Eagle’s Medium (DMEM)/F-12 media (1:1) (Thermo Fisher) containing 10% heat-inactivated fetal bovine serum and 1% penicillin/streptomycin in an incubator (37 ℃, 5% CO_2_) as previously described (53). When the cells reached 70 to 80% confluency, they were transfected with plasmids for ADRB1 or PSD-95 using polyethylenimine (Polysciences). After 24 h, cells were harvested for further analysis. For SH-SY5Y differentiation, cells were cultured in a 4-well chamber slide (Nunc™ Lab-Tek™ II, Thermo Scientific) with DMEM/F-12 media. The next day, cells were treated with 5 µM RA to induce differentiation for 5 d, and the medium was changed every other day. At day 6, the cells were transfected with plasmids. After 24 and 48 h, the cells were fixed for immunofluorescent staining.

### Statistical Analysis.

All statistical analyses and graph preparation were performed using GraphPad Prism 9. Data are shown as means ± SEM unless otherwise explicitly stated. Statistical differences between experimental groups were assessed by two-tailed Student’s *t* test for comparing two groups, paired *t* test for comparing treatment groups vs. control group, one-way ANOVA with post-hoc multiple comparisons when comparing multiple groups, and two-way ANOVA with post-hoc multiple comparisons for multifactorial analyses. All statistical analysis results are in Dataset S1.

## Supplementary Material

Appendix 01 (PDF)

Dataset S01 (XLSX)

## Data Availability

Anonymized RNA-seq data have been deposited in NCBI-GEO (GSE205558). All study data are included in the article and/or supporting information.
