# Supplementary material for: Mutant β1-adrenergic receptor improves REM sleep and ameliorates tau accumulation in a mouse model of tauopathy
Source: Proc Natl Acad Sci U S A. 2023 Apr 4;120(15):e2221686120. doi: 10.1073/pnas.2221686120 (PMC10104526; doi:10.1073/pnas.2221686120)
Supplement: Supplementary file 1 — Appendix 01 (PDF) [file pnas.2221686120.sapp.pdf]

Supplementary Materials

Mutant β1-adrenergic receptor improves REM sleep and ameliorates tau accumulation in a mouse model of tauopathy

Qing Dong, Louis J. Ptáček* and Ying-Hui Fu*

*Correspondence to: Ying-Hui.Fu@ucsf.edu (Y-H.F.); ljp@ucsf.edu (L.J.P.)

This file includes:

Fig. S1 to S5 for multiple supplementary figures

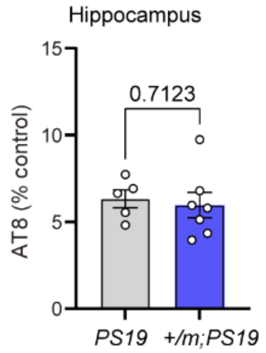

Fig. S1. P-tau staining in the hippocampus of *Adrb1-A187V;PS19* and *PS19* mice.

The tau pathology indicated by staining with AT8 antibody in the hippocampus of 8-month-old *Adrb1-A187V;PS19* and *PS19* mice. *n* = 5-7 mice per group. Data are means ± SEM; *P* values represent an unpaired two-tailed *t*-test.

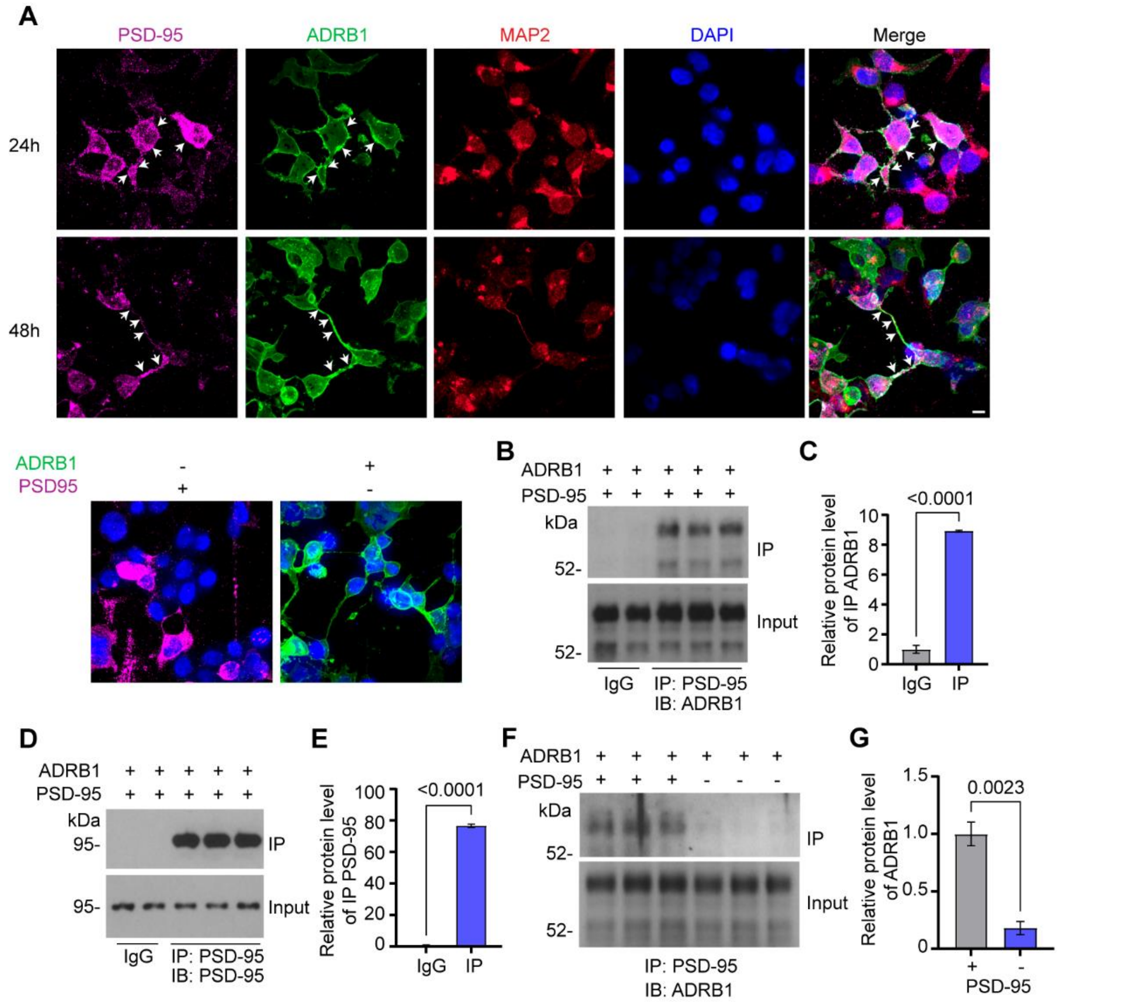

Fig. S2. Confirmation of the interaction between ADRB1 and PSD-95.

(*A*) Representative images showing the co-localization of PSD-95 and ADRB1 in the differentiated SH-SY5Y cells 24 and 48 hours after the transfection. Scale bar, 10 μm (top and middle). There is no cross immunoreactivity between ADRB1 and PSD-95 antibodies (bottom). (*B-E*) IP with PSD-95 antibody revealed the positive signals of both PSD-95 and ADRB1, but no obvious signals were detected using IgG. *n* = 2 or *n* = 3 biological replicates. Data are means ± SEM; *P* values represent an unpaired *t*-test (*C* and *E*). (*F* and *G*) The ADRB1 can only be pulled-down by the PSD-95 antibody in the cells co-transfected with ADRB1 and PSD-95, but not in the cells only expressing ADRB1. *n* = 3 biological replicates. Data are means ± SEM; *P* values represent an unpaired *t*-test (*G*).

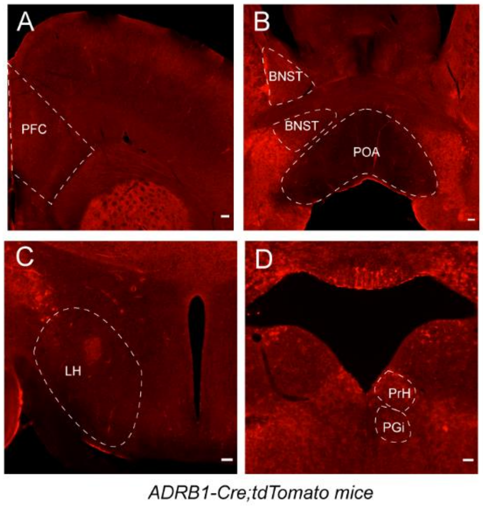

Fig. S3. No obvious ADRB1 positive signal in the non-CeA areas for inputs to the LC.

(*A*-*D*) Detecting the ADRB1$^+$ neurons in the prefrontal cortex (PFC) (*A*), bed nucleus of the stria terminalis (BNST), preoptic area (POA) (*B*), lateral hypothalamus (LH) (*C*), prepositus hypoglossus (PrH) and paragigantocellularis (PGi) (*D*) in the *ADRB1-Cre;tdTomato* mice. Scale bar, 100 μm.

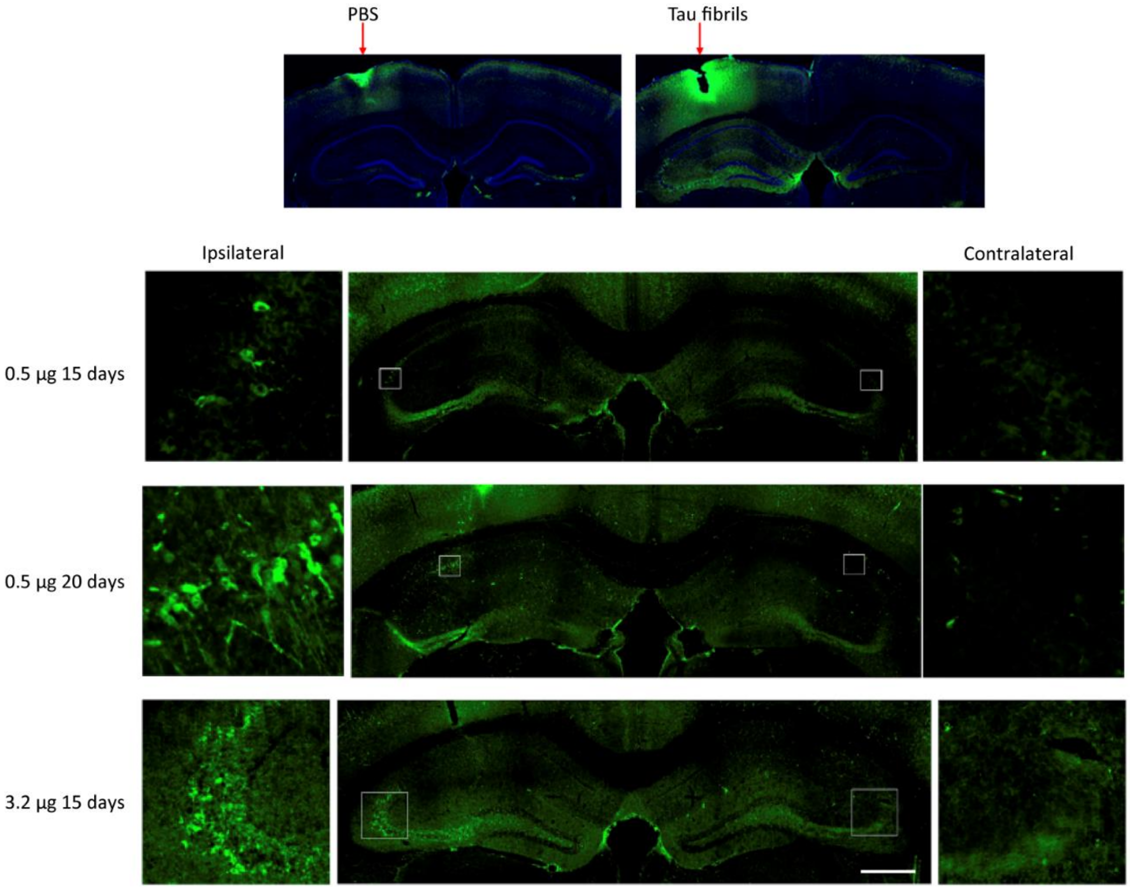

Fig.S4. Tau fibril seeds in the brain in a dose- and time-dependent manner.

Different dose of tau fibril or PSB was unilaterally injected into the hippocampus of *PS19* mice. 15 day or 20 day of post injection, the seeding of tau was detected. *n* = 2 or 5 mice per group. Scale bar, 20 µm.

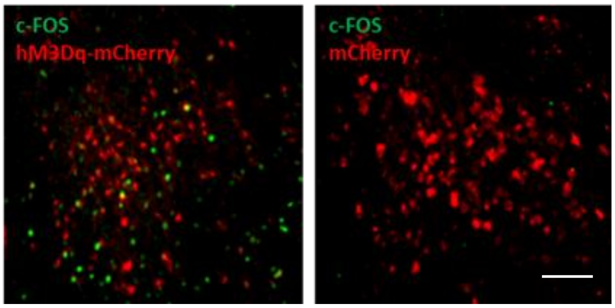

Fig. S5. The level of c-FOS was increased in the CeA after CNO treatment.

After 6-hour treatment of CNO, the expression of c-FOS was increased in the CeA of the mice injected with hM3Dq-mCherry but not mCherry. *n* = 4 mice for experimental group; *n* = 2 mice for control group. Scale bar, 100 µm.
